# Gigantism: microsurgical treatment by transsphenoidal approach and prognostic factors

**DOI:** 10.1007/s11102-022-01286-0

**Published:** 2022-11-06

**Authors:** María García-Uría Santos, Cecilia Fernández Mateos, Tomás Lucas Morante, José García-Uría

**Affiliations:** 1 Service of Emergency Medicine. Clínica, Universidad de Navarra, Madrid, Spain; 2 Service of Neurosurgery, Hospital Puerta de Hierro Majadahonda, Madrid, Spain; 3 Service of Endocrinology, Hospital Puerta de Hierro Majadahonda, Madrid, Spain

**Keywords:** Gigantism, Acromegaly, Pituitary adenoma, Microsurgery, Growth hormone

## Abstract

**Purpose:**

We present the results of transsphenoidal microsurgical treatment in 14 patients with gigantism. The influence on the prognosis of factors such as the tumor size and preoperative levels of GH and IGF-1 is also quantified.

**Materials and methods:**

The patients, operated between 1982 and 2004, were reviewed retrospectively in June 2022. All patients had complete endocrinological studies in the preoperative period and a postoperative control between 6 days and 3 weeks. Follow-up has been supported with annual check-ups between 3 and 31 years. We have compared the preoperative levels of GH and IGF-1 of these patients with the levels of a series of acromegalic patients operated on in the same Center.

**Results:**

In this series there were 4 women and 10 men. The age ranged between 14 and 21 years. In 6 patients, postoperative hormone levels achieved the disease control criteria (42.8%). The CT/MRI studies revealed the existence of invasive tumors in 10 of the patients (71.4%). Postoperative CT/MRI showed no tumor tissue in 3 patients but in 7 patients there were tumor remains. The remaining 4 patients had abnormal images although not considered as tumor. A statistical comparison of preoperative serum GH and IGF-1 levels in patients with gigantism and patients with acromegaly showed a significant elevation in the former.

**Conclusion:**

Pituitary adenomas that cause gigantism are generally large and invasive, which makes them difficult to cure. High preoperative levels of GH and IGF-1 are also factors that decrease remission.

## Introduction

The clinical picture of gigantism is well known, and it is caused by an excess of growth hormone (GH) during childhood, before epiphyseal closure, causing disproportionate linear growth. The clinical and genetic characteristics of this disease have been thoroughly analyzed [1–3].

GH hypersecretion in adults produces the clinical manifestations of acromegaly. As gigantism and acromegaly have a common origin, both pathologies share some clinical features. Approximately 10% of acromegalic patients are gigantic in height and acromegalic characteristics can be seen in most teenagers with gigantism [1].

An excessive secretion of GH is mainly caused by a pituitary adenoma and surgical treatment is the first therapeutic choice. Its main goal is to achieve the normalization of GH and IGF-1 levels through a selective resection of the adenoma.

Gigantism is a rare disease and objective data on the results of its surgical treatment are scarce. Most information comes from the analysis of series of pituitary adenomas in childhood, of which approximately 5–25% produce hypersecretion of GH and not always gigantism [4–7].

There are few series of patients with gigantism treated by transsphenoidal microsurgery that have been published [8, 9]. In those series, a significant percentage of huge GH secreting adenomas with invasive characteristics were collected. The influence of tumor size and peripheral invasion as the most important prognostic factors in surgical results of hypersecreting adenomas is well known. Under these circumstances, the chances of surgical cure are estimated between 13% [3, 9] and 23% [8]. These cure ratings are much lower than those usually obtained in acromegalic patients. However, in those mentioned articles, the post-surgical long-term evolution has not been studied. A specific quantification of GH and IGF-1 levels and their possible influence on the prognosis of surgical treatment has not been carried out.

The aim of this work is to show the results of transsphenoidal microsurgical treatment in 14 patients with gigantism. The evolution of the initial results will be quantified by long-term postoperative monitoring of GH and IGF-1 levels. In addition, the preoperative levels of GH and IGF-1 and their prognostic values are analyzed.

## Materials and methods

Between 1982 and 2004, 14 patients with clinical gigantism due to pituitary adenomas with hypersecretion of GH were surgically treated at the Puerta de Hierro Clinic in Madrid.

The patients were studied endocrinologically by the same endocrinologist and all patients had GH, IGF-1, PRL, ACTH, TSH, LH and FSH studies performed in the Center's laboratory in both pre- and postoperative periods. Laboratory techniques include radioimmunoassay (RIA) and immunoradiometric assay (IRMA) for the study of GH and IGF-1. In 3 patients, the preoperative/initial postoperative study did not include IGF-1 values ​​because they were treated before having IGF-1 levels available at the Center.

The most recent review of the hormonal and radiological data of these patients was performed in June 2022, based on the data collected in their medical records. There were 8 patients with a follow-up period greater than 10 years and although the follow-up of the remaining 6 patients is diverse, all of them had records of at least 3 years.

The endocrinological postoperative follow-up studies of three patients has been carried out in our Center until year 2021–22. The other 11 patients have been followed in other centers since they came from cities other than Madrid. However, those patients were followed in our Center for a postoperative period from 3 to 12 years before they began follow-up control in other hospitals. Even though 9 of these patients were reached by phone in June 2022, we established the follow-up time in relation to the last test carried out in our Center considering that verbal information didn’t modify the known results.

The preoperative radiological diagnosis of pituitary adenoma was proved by a CT study in 2 patients operated on before 1986 and with an MRI study in the other 12 patients. All 14 patients had postoperative MRI studies that have allowed us the quantification of their evolution.

According to the tumor size, patients were classified into four grades. Grade I: microadenoma; Grade II: non-invasive macro adenomas; Grade III: macro adenomas with moderate invasions; Grade IV: highly invasive giant adenomas.

The patients underwent surgery with the aim of performing a selective resection of the GH-secreting pituitary adenoma. All of them were treated by the same surgeon who used a similar sublabial, transsphenoidal microsurgical approach.

Surgical outcomes were quantified by postoperative GH and IGF-1 levels determined between six days and three weeks after the intervention. Disease control was defined at that time by GH levels of less than 2 ng/ml with a decrease of less than 1 ng/ml after oral glucose overload (OGTT) and with normal IGF-1 levels according to age and sex.

In order to establish the relative value of excessive GH and IGF-1 levels in gigantism related to hypersecretion levels in patients with acromegaly, we have compared both hormone levels in our 14 patients with gigantism to the same hormone levels in a series of surgically treated acromegalic patients with a similar technique in the same Center and quantified with the same laboratory test. These adults with acromegaly had been subjects in an earlier study [10]. A descriptive analysis has been performed with absolute and relative frequencies to describe the categorical (qualitative) variables through the mean and standard deviation or median and 25^th^ and 75^th^ percentiles in the quantitative variables. The Wilcoxon tests and the Mann–Whitney and Kruskall-Wallis U tests were used in the analysis. The software used for the statistical analysis of the data was the STATA version 16 program, with an established level of significance of p < 0.05.

## Results

### General characteristics

In this series of patients with gigantism, there were 4 women and 10 men. The age at the time of the intervention ranged between 14 and 21 years with a mean of 18.2 years. The time of evolution of the clinical picture at the time of diagnosis varied between 1 and 15 years, with a mean of 3.38 years. (Table 1).

**Table 1:** Universal characteristics of the patients

Patients	Age (years)		Sex		Evolution (years)
	Mean	Range	Women	Men	
14	18.2	14–21	4 (28.57%)	10 (71.42%)	3.3

There was galactorrhea in one patient and hypertension in another one. In addition, visual field defects were detected in 3 of the 14 patients (21.4%).

### Hormonal values

Preoperative GH levels ranged from 2 ng/ml to 162 ng/ml with a mean of 45.4 ng/ml. Preoperative IGF-1 levels ranged from 522 ng/ml to 1544.4 ng/ml with a mean of 833.5 ng/ml.

There was LH and FSH deficiency at the time of diagnosis in 4 of the 14 patients and after surgery only one of them reached normal LH and FSH values. As for the remaining ten patients, although they presented preoperative GH hypersecretion, the rest of the pituitary function remained normal.

As we mentioned before, there were 4 female patients and 10 male patients. One woman with a grade I tumor achieved normal GH levels after surgery, and she also normalized preoperative gonadotropins deficit. The remaining three women were treated with radiotherapy and while two patients developed panhypopituitarism, the third patient showed decreased LH and FSH levels.

Three of the 10 male patients had a preoperative decrease of LH and FSH levels and none improved with surgery. Four patients were treated with radiotherapy; three developed panhypopituitarism while the other patient developed a deficiency of LH, FSH and TSH levels.

### Radiological imaging: size and invasion

The CT/MRI studies revealed the existence of invasive tumors in 10 of the patients (71.4%). In 5 patients, the tumor was classified as Grade III and the other 5 patients presented with a Grade IV adenoma. There were 2 patients with a Grade I microadenoma and 2 patients with Grade II macroadenoma.

### Surgical results

The results quantification of the surgical treatment was carried out through the changes in GH and IGF-1 levels determined in the immediate postoperative period according to current remission criteria. (Table 2).

**Table 2:** Preoperative and postoperative hormone levels. Patients in remission according to tumor grade

MRI preop		GH (ng/ml)		IGF-1 (ng/ml)		Remission	MRI Postop	Follow-up (years)	Additional treatment
	Patients	Preop	Postop	Preop	Postop (NR)*	(YES/NO)			
Grade I	2	7.1	0.3	1224	357 (71–234)	YES	Normal	12	–
		12.2	0.4	522	197 (190–429)	YES	Normal	4	–
Grade II	2	19	6.6	–-	–-	NO	Tumor	22	RT^*^
		68	2.7	1000	365 (71–234)	NO	No normal	4	SSA^*^
Grade III	5	39.4	0.8	–-		YES	No normal	4	–
		47	5	1544	408 (117–323)	NO	Tumor	16	RT^*^
		162.4	6.4	891	730 (173–414)	NO	Tumor	7	RT^*^
		61.6	13.4	1227	402 (177–507)	NO	Tumor	6	RT^*^
		32.2	0.6	1269	480 (127–323)	YES	Normal	5	–
Grade IV	5	36	0.3	–-	–-	YES	No normal	31	–
		32	31	900	840 (173–323)	NO	Tumor	12	RT^*^
		73	100	919	1004 (117–323)	NO	Tumor	3	RT^*^
		68	2.7	1000	365 (173–414)	NO	Tumor	9	RT^*^
		2	0.5	1089	470 (245–300)	YES	No normal	9	–
Total	**14**					**6 (42.8%)**			

^*^NR normal ranges, *RT* radiotherapy, *SSA* somatostatin analogue therapy

In 6 of the 14 patients, postoperative hormone levels met accepted disease control criteria at the time of surgery. All six patients achieved immediate postoperative GH levels below 1 ng/ml. However, in three patients, IGF-1 needed several weeks to reach values ​​within the normal range. Even so, it is important to highlight that during the follow-up of the six patients, the hormonal studies performed annually did not show recurrence of hypersecretion of GH nor IGF-1.

The CT/MRI studies in the initial postoperative period showed no tumor tissue in three patients: two patients with microadenoma and one patient with a grade III adenoma. In seven patients there were tumor remains and four patients presented with abnormal images suggestive of intrasellar remains related to the intervention itself, but without detecting image of adenoma. In those patients with abnormal images, postoperative hormone levels in three patients were normal.

In the group of patients not cured after surgical treatment (8 patients), several types of radiotherapy were used in seven of them. This treatment was effective in controlling hormonal hypersecretion in 4 of them. (Table 3). On the other hand, there was a patient with abnormal hormone levels who received treatment with somatostatin analogs (SSA).

**Table 3:** Remission rates in patients after receiving postoperative adjuvant radiotherapy according to tumor grade

MRI preop	Patients	Surgical Failure (n)	RT	Remission	Follow-up (years)
Grade I	2	0	–	–	–
Grade II	2	2	1	1	22
Grade III	5	3	3	2	6 16
Grade IV	5	3	3	1	12
**Total**	**14**	**8**	**7**	**4**	

### Follow-up

The follow-up control was greater than three years in all the patients, with a mean of 10.2 years. Follow-up was longer than 5 years in 10 patients, longer than 10 years in 5 patients, and longer than 15 years in 3 patients.

### Complications

There was no mortality in this series. No postoperative CSF fistula, meningitis, or functional involvement of cranial nerves appeared. No patient reported symptoms of diabetes insipidus three months after the intervention.

For all the information mentioned above, the rate of complications produced by the intervention can be considered as satisfactory.

### Comparison of hormone levels between gigantism and acromegaly

A statistical comparison of preoperative serum GH levels in the 14 patients with gigantism and preoperative GH levels in 460 acromegalic patients that underwent surgery between 1982 and 2004 in the same Center, demonstrates a significant elevation of GH levels in patients with gigantism. (Table 4).

**Table 4:** Baseline GH values ​​prior to surgery

	Patients	GH (ng/ml)		
		p25	p50	p75
Gigantism	14	19	36.2	68
Acromegaly	460	6.95	15.4	34.7

Comparison between 14 patients with gigantism and 460 acromegalic adults operated on in the same period

p = 0.017001

A comparison between preoperative IGF-1 levels in 11 giants and IGF-1 levels in a population of 363 adults with acromegaly operated on at the same Center also demonstrates much higher levels of IGF-1 hypersecretion in giants, with statistical significance. (Table 5).

**Table 5:** Baseline IGF-1 values ​​prior to surgery

	Patients	IGF-1 (ng/ml)		
		p25	p50	p75
Gigantism	11	902	1008	1225.5
Acromegaly	363	610	807	1106

Comparison of 11 patients with gigantism with 363 acromegalic adults operated on in the same period

p = 0.0486

## Discussion

Gigantism is considered a rare disease and there is evidence in the literature that supports (likely other pituitary adenomas in childhood) that adenomas in giants are usually large and have invasive characteristics [7]. The appearance of visual disturbances [8] is not uncommon and a predominance of the disease in males has been published [2, 8, 9], although other articles report higher frequency in women [7]. These facts are also present in this series.

The results of surgical treatment in patients with gigantism are quantified based on the changes introduced by the intervention, the hormonal levels and the normalization of the radiological imaging that shows the resection of the adenoma.

Specific endocrinological remission criteria for gigantism have not been established. We have always used the current criteria for acromegaly even though the remission criteria for acromegaly in relation to postoperative hormonal values have evolved in recent decades [11–14]. Our patients were operated on at a time when the criteria considered for a reliable cure were those mentioned before in Materials and Methods, and these were the ones considered for the clinical management of the patient at that time as well as for making the decision of using other adjuvant therapy in those cases in which it was considered necessary.

In this series, six patients (42.8%) achieved the remission criteria used at that time, four of which were large invasive adenomas. These results are better than those reported by some authors in the surgical treatment of gigantism [8, 9], although they do not seem as good when compared with series of acromegalic patients treated with microsurgery [15–17].

Tumor size and invasion seem to play a significant role in these results. In addition, the consistency of the tumor tissue and the anatomical characteristics related to forms of invasion, particularly in the cavernous sinuses, are also important as they can facilitate the complete resection of giant tumors.

To our knowledge no one has ever compared hormone hypersecretion levels between patients with gigantism and patients with acromegaly. This series suggests that adenomas of patients with gigantism secrete more GH and more IGF-1 levels than in patients with acromegaly. As well as size and invasion, higher GH levels contributes to reduce the possibilities of surgery [18] and probably other therapies available to us.

The six patients that achieved remission in this series had GH preoperative levels below 40 ng/ml.

The results of the surgical intervention on the tumor mass are clear in those cases in which the MRI shows the absolute normality of the sella content (even with the gland perfectly visible) and in those cases in which the resection is incomplete and tumor remains can be viewed. However, in four of our patients the MR image was abnormal although without evident tumor tissue. These are cases in which the sella turcica is occupied by material that has been defined as post-surgical changes in relation to possible collections of blood and scar tissue. It is interesting to know that three of those four patients achieved normal GH and IGF-1 levels.

Although the use of radiotherapy in young patients carries an added risk the therapeutic results obtained in this series can be considered as satisfactory. Disease control was achieved in four of the seven patients who were treated with this therapy, results that agree with other authors [8, 9, 19]. On the other hand, one patient in whom the intervention failed was successfully controlled with medication (somatostatin analogs).

The follow-up time of the patients in this series gives a perspective to the results. To our knowledge, there are no published data with an evolutionary follow-up of more than two years [7]. Our data suggest that if patients manage to achieve the criteria for endocrinological cure, disease control could be maintained for years without a relapse. This data supports the predictive value of the initial postoperative GH levels as referenced by other authors [18].

The risks of transsphenoidal microsurgical resection of pituitary adenomas are not trivial [10], although in this series the complications have remained within a very satisfactory range. Nevertheless, it should be considered that as the number of patients is very low, a clear significance cannot be assumed at this point.

In recent years there has been a trend towards the use of endoscopic techniques for the treatment of tumors in the sella turcica [20,21]. Nowadays, endoscopic surgery has not been shown to be superior to transsphenoidal microsurgery in the treatment of GH hypersecreting adenomas [10], although there is no reason to consider that results of endoscopy may be very different from those obtained by microsurgical approach [22]. There are references of its efficacy in treatment of pituitary adenomas in young patients [23].

## Conclusions

A series of 14 patients with gigantism due to pituitary adenoma operated on with transsphenoidal microsurgery is presented. Surgical intervention achieved control of the disease in 42.8% of patients, representing a lower rate than the one usually managed in adults with acromegaly. This prognosis has been mainly related to three different factors: the size and characteristics of the adenoma (71.4% invasive) and the increased GH and IGF-1 levels, statistically higher than those produced in acromegaly.

The evolutionary follow-up from 3 to 31 years with a mean of 10.2 years shows that in controlled patients there have been no recurrences of the hormonal hypersecretion and supports the predictive value of the initial postoperative GH levels. However, more studies are needed to obtain more consistent information.

## References

[1] Eugster EA, Pescovitz OH (1999). Gigantism. J Clin Endocrinol Metab.

[2] Chentli F, Azzoug S, Amani Mel A (2012). Etiologies and clinical presentation of gigantism in Algeria. Horm Res Paediatr.

[3] Rostomyan L, Daly AF, Petrossians P (2015). Clinical and genetic characterization of pituitary gigantism: an international collaborative study in 208 patients. Endocr Relat Cancer.

[4] Dyer EH, Civit T, Visot A (1994). Transsphenoidal surgery for pituitary adenomas in children. Neurosurgery.

[5] Barzaghi LR, Losa M, Capitanio JF (2019). Pediatric pituitary adenomas: early and long-term surgical outcome in a series of 85 consecutive patients. Neurosurgery.

[6] Locatelli D, Veiceschi P, Castelnuovo P (2019). Transsphenoidal surgery for pituitary adenomas in pediatric patients: a multicentric retrospective study. Childs Nerv Syst.

[7] Jayant SS, Pal R, Rai A (2022). Paediatric pituitary adenomas: clinical presentation, biochemical profile and long-term prognosis. Neurol India.

[8] Creo AL, Lteif AN (2016). Pituitary gigantism: a retrospective case series. J Pediatr Endocrinol Metab.

[9] García WR, Cortes HT, Romero AF (2019). Pituitary gigantism: a case series from Hospital de San José (Bogotá, Colombia). Arch Endocrinol Metab.

[10] Fernández Mateos C, García-Uria M, Morante TL (2017). Acromegaly: surgical results in 548 patients. Pituitary.

[11] Giustina A, Barkan A, Casanueva FF (2000). Criteria for cure of acromegaly: a consensus statement. J Clin Endocrinol Metab.

[12] Melmed S, Bronstein MD, Chanson P (2018). A Consensus Statement on acromegaly therapeutic outcomes. Nat Rev Endocrinol.

[13] Giustina A, Barkan A, Beckers A (2020). A consensus on the diagnosis and treatment of acromegaly comorbidities: an update. J Clin Endocrinol Metab.

[14] Fleseriu M, Biller BMK, Freda PU (2021). A Pituitary Society update to acromegaly management guidelines. Pituitary.

[15] Nomikos P, Buchfelder M, Fahlbusch R (2005). The outcome of surgery in 668 patients with acromegaly using current criteria of biochemical 'cure'. Eur J Endocrinol.

[16] Ludecke DK, Abe T (2006). Transsphenoidal microsurgery for newly diagnosed acromegaly: a personal view after more than 1000 operations. Neuroendocrinology.

[17] Buchfelder M, Schlaffer SM (2017). The surgical treatment of acromegaly. Pituitary.

[18] Agrawal N, Ioachimescu AG (2020). Prognostic factors of biochemical remission after transsphenoidal surgery for acromegaly: a structured review. Pituitary.

[19] Pandey P, Ojha BK, Mahapatra AK (2005). Pediatric pituitary adenoma: a series of 42 patients. J Clin Neurosci.

[20] Anik I, Cabuk B, Gokbel A (2017). Endoscopic transsphenoidal approach for acromegaly with remission rates in 401 patients: 2010 consensus criteria. World Neurosurg.

[21] Babu H, Ortega A, Nuno M (2017). Long-term endocrine outcomes following endoscopic endonasal transsphenoidal surgery for acromegaly and associated prognostic factors. Neurosurgery.

[22] Chen CJ, Ironside N, Pomeraniec IJ (2017). Microsurgical versus endoscopic transsphenoidal resection for acromegaly: a systematic review of outcomes and complications. Acta Neurochir (Wien).

[23] Dhandapani S, Narayanan R, Jayant SS (2021). Endonasal endoscopic versus microscopic transsphenoidal surgery in pituitary tumors among the young: A comparative study & meta-analysis. Clin Neurol Neurosurg.

